# Forecasting *Culex pipiens/restuans* abundance in Ontario, Canada using surveillance and weather data

**DOI:** 10.1093/jme/tjag159

**Published:** 2026-11-05

**Authors:** Zhaowan Li, Ziwang Deng, Huaiping Zhu

**Affiliations:** Department of Mathematics and Statistics, Laboratory of Mathematical Parallel Systems (LAMPS) and Centre of Diseases Modeling (CDM), York University, Toronto, ON, Canada; Department of Mathematics and Statistics, Laboratory of Mathematical Parallel Systems (LAMPS) and Centre of Diseases Modeling (CDM), York University, Toronto, ON, Canada; Department of Mathematics and Statistics, Laboratory of Mathematical Parallel Systems (LAMPS) and Centre of Diseases Modeling (CDM), York University, Toronto, ON, Canada

**Keywords:** *Culex pipiens*/*restuans*, mosquito abundance, weather variables, machine learning, week-ahead forecasting

## Abstract

Reliable short-term forecasting of vector abundance is critical for guiding localized mosquito control, yet week-ahead prediction at the Public Health Unit (PHU) scale remains challenging. We developed and evaluated a week-ahead forecasting framework for *Culex pipiens/restuans* abundance using 22 years of routine surveillance and meteorological data from 34 PHUs across Ontario, Canada. Based on a subset of predictors selected through feature-selection techniques, models integrating recent mosquito abundance and lagged weather variables were evaluated with multiple regression-based approaches, including linear, neural network, and random-forest regression, under random, temporal, and spatial validation schemes. Under cross-validation, top-performing models explained approximately 69% of the variance in weekly mosquito abundance. In independent testing on 2022 to 2023 data, the best-performing model achieved a 14.4% reduction in RMSE and an absolute increase in $R^{2}$ of 0.124 relative to the persistence baseline. Across validation schemes, predictive performance was driven primarily by recent mosquito abundance history, whereas recent weather conditions provided additional gains, with contributions attenuating at longer lags. Increasing predictor complexity yielded diminishing returns for week-ahead forecasting. Consistent performance across validation schemes indicates model robustness. Overall, these results demonstrate a parsimonious approach for routine short-term forecasting at the PHU scale, with potential applications in surveillance, resource allocation, and targeted mosquito control.

## Introduction

*Culex pipiens Linnaeus*, 1758 and *Culex restuans Theobald*, 1901 (Diptera: Culicidae) are the primary vectors of West Nile virus (WNV) in eastern North America, posing substantial public health risks (Yoo et al. 2016, Centers for Disease Control and Prevention 2024). Forecasting of *Cx. pipiens/restuans* abundance can support mosquito-borne disease prevention by improving situational awareness, informing the timing and targeting of vector control interventions, and helping public health authorities allocate surveillance resources more efficiently (Bakran-Lebl et al. 2023, Ferraguti et al. 2024, Steindorf et al. 2025). Because mosquito abundance responds strongly to environmental conditions and exhibits pronounced seasonal and interannual variability, reliable short-term forecasting remains both operationally important and methodologically challenging (Talbot et al. 2023).

Significant progress has been made in modeling *Cx. pipiens/restuans* abundance in Ontario. Early work in the Peel Region used gamma-generalized linear models to characterize associations between weather conditions and mosquito abundance (Wang et al. 2011). Subsequent studies developed increasingly mechanistic representations of mosquito population dynamics, including ordinary differential equation models with temperature-dependent development, mortality, and diapause functions (Yu et al. 2018), process-based models integrating temperature, hydrological conditions, and laboratory-derived parameters to capture nonlinear interactions between climate and aquatic habitats (Shutt et al. 2022), and age-structured partial differential equation models that explicitly represent within-stage population structure and reproduce observed abundance patterns (Frantz et al. 2024). Collectively, these studies have advanced understanding of the environmental controls on *Cx. pipiens/restuans* populations and strengthened the broader capacity to predict mosquito abundance.

Alongside these mechanistic developments, machine learning methods have emerged as flexible tools (Yang et al. 2024) for modeling ecological systems characterized by nonlinear responses (Lek and Guégan 1999, Olden et al. 2008, Hoyos et al. 2021), lagged effects (Lebl et al. 2013, Hoyos et al. 2021), and complex interactions among predictors (Cutler et al. 2007, Ong et al. 2023). A growing body of research has applied machine learning to mosquito abundance prediction in a range of settings. For example, Chen et al. (2019) used supervised learning algorithms to verify that landscape heterogeneity and socioeconomic gradients were associated with mosquito abundance in urban environments. Tsantalidou et al. (2021) subsequently developed a novel framework that combines multi-source Earth observation data with gradient boosting to generate genus-agnostic abundance predictions across diverse European landscapes. More recently, Talbot et al. (2023) reported that random forest models outperformed traditional regression approaches when applied to long-term mosquito surveillance data in Ontario. Similar methodological advantages have also been reported in related hematophagous dipteran systems; for example, De Klerk et al. (2024) used a 26-year dataset on *Culicoides spp*. in South Africa to show that ensemble methods, particularly random forest, can capture complex abundance patterns over broad spatial and temporal scales. Collectively, these studies indicate that machine learning methods can provide effective tools for predicting mosquito abundance from surveillance-related data.

Despite these advances, important gaps remain in the mosquito-focused machine learning literature. Many published machine learning studies rely on relatively short time series, which can limit the range of lagged meteorological predictors that can be incorporated without increasing the risk of overfitting and may reduce the ability to capture interannual climatic variability and longer-term population dynamics. In addition, most existing models have been developed for specific study areas or research contexts and do not provide a standardized approach for generating consistent Public Health Unit (PHU)-specific forecasts at a provincial scale using routinely available surveillance and weather inputs. Although mechanistic models provide valuable ecological insight, their routine operational use may be constrained by structural assumptions, parameter uncertainty, and the need for detailed biological inputs that are not always available in surveillance settings. There is therefore a need for a scalable forecasting framework that can integrate long-term surveillance data with routinely available weather information to deliver robust, interpretable, and operationally useful week-ahead predictions.

To address this need, we developed and validated a week-ahead forecasting approach for *Cx. pipiens/restuans* abundance using 22 years of surveillance data from 34 Ontario PHUs linked to PHU-aggregated meteorological covariates. Our objective was to assess whether a parsimonious data-driven approach could generate reliable short-term forecasts using routinely available surveillance and weather data. To this end, we combined recent mosquito abundance history with seven meteorological variables across multiple temporal lags, applied three complementary feature-selection methods, and compared three predictive model classes: stepwise multiple linear regression, feedforward neural networks, and random forest regression. Model performance was evaluated using random, temporal, and spatial validation, followed by independent testing on 2022 to 2023 data. The principal contribution of this study is the delivery of a standardized, PHU-specific forecasting framework that remains operationally feasible and grounded in long-term data, thereby strengthening preparedness for mosquito-borne disease risk management in Ontario. For navigational clarity, all abbreviations used throughout the text are defined in Appendix Table A1.

**Table 1.** Predictive performance of SMLR, NN, and RFR models under 10-fold cross-validation using the 2002 to 2021 development dataset. RMSE and $R^{2}$ were calculated on the rescaled log-transformed WMA scale

ID	Predictors	RMSE				$R^{2}$			
		SMLR	NN	RFR	Ave	SMLR	NN	RFR	Ave
**1**	A	0.146	**0.145**	0.149	0.147	0.602	**0.604**	0.586	0.597
**2**	B	0.194	0.192	**0.190**	0.192	0.299	0.315	**0.326**	0.313
**3**	C	0.185	0.183	**0.179**	0.182	0.361	0.371	**0.404**	0.379
**4**	AB	0.133	**0.132**	0.133	0.132	0.672	**0.675**	0.670	0.672
**5**	AC	0.134	0.133	**0.133**	0.133	0.665	0.669	**0.671**	0.668
**6**	BC	0.180	0.178	**0.170**	0.176	0.397	0.407	**0.457**	0.420
**7**	CD	0.181	0.180	**0.172**	0.178	0.386	0.393	**0.445**	0.408
**8**	ABC	0.131	0.130	**0.130**	0.130	0.681	0.684	**0.685**	0.683
**9**	ACD	0.132	0.131	**0.131**	0.132	0.674	0.675	**0.678**	0.676
**10**	BCD	0.176	0.174	**0.166**	0.172	0.424	0.432	**0.485**	0.447
**11**	ABCD	0.129	0.129	**0.129**	0.129	0.687	0.687	**0.689**	0.688
**12**	E	0.129	0.130	**0.129**	0.129	0.691	0.687	**0.691**	0.689
**13**	F	0.129	0.131	**0.129**	0.130	0.689	0.687	**0.692**	0.689
**14**	G	0.130	0.131	**0.129**	0.130	0.684	0.678	**0.689**	0.683
**15**	Average	0.151	0.150	**0.148**	0.149	0.565	0.569	**0.583**	0.572

“Ave” denotes the mean performance across the three model types. Predictor sets are defined as A, lagged WMA; B, concurrent weather; C, weather at lags 1 to 2; D, weather at lags 4 to 6; and E, F, and G, predictor sets selected by SR, LASSO, and RF, respectively. Combined labels indicate the union of the corresponding predictor groups. Within each predictor set (row), bold values indicate the best-performing model (lowest RMSE and highest *R²*).

## Materials and Methods

### Surveillance Program

Mosquito surveillance in Ontario was initiated in 2001 by the Ministry of Health and Long-Term Care. Across the province, local PHUs followed standardized trapping protocols using Centers for Disease Control miniature light traps baited with CO_2_ and light to attract host-seeking adult female mosquitoes. Trapping was conducted weekly, generally from mid-June to early October (typically epidemiological weeks 24 to 39), although official surveillance records span approximately weeks 18 to 43. Collected mosquitoes were identified to species level, counted, and tested for WNV. Because *Cx. pipiens* and *Cx. restuans* are morphologically similar, they are typically grouped and reported as a combined taxon. For reference, the 34 PHUs included in this study are listed in Table A2 (Appendix Public Health Ontario 2025).

**Table 2.** Predictive performance of SMLR, NN, and RFR models on the independent 2022 to 2023 test set (PHU-week observations). RMSE and *R²* values are reported on the rescaled log-transformed WMA: ln(1 + WMA) rescaled to [0,1]; corresponding RMSE values computed on the original WMA scale are provided in parentheses. Predictor-set definitions are the same as in Table 1

Predictor	RMSE (SMLR)	RMSE (NN)	RMSE (RFR)	*R*² (SMLR)	*R*² (NN)	*R²* (RFR)
**AB**	0.143 (6.2)	0.143 (6.1)	0.145 (6.1)	0.663	0.665	0.656
**AC**	0.139 (5.8)	0.142 (6.1)	0.142 (6.0)	0.685	0.668	0.668
**ABC**	0.140 (6.0)	0.141 (6.0)	0.141 (6.1)	0.679	0.676	0.672
**ABCD**	0.141 (6.0)	0.141 (6.1)	0.143 (6.3)	0.674	0.671	0.664
**E**	0.142 (5.9)	0.139 (5.9)	0.142 (6.3)	0.667	0.682	0.670
**F**	0.140 (5.9)	0.137 (5.9)	0.145 (6.5)	0.678	0.694	0.653
**G**	0.141 (6.0)	0.141 (6.2)	0.147 (6.5)	0.674	0.671	0.647

Ontario’s provincial mosquito surveillance program generally operates each active trap for one night per week during the surveillance season. More than 300 traps have historically been operated across the province from June through October (Public Health Ontario 2017). However, the number of active traps varies among PHUs and may also vary among years and weeks. For example, an analysis of 14 Greater Golden Horseshoe PHUs during 2002 to 2016 reported approximately 2 to 85 active traps per week, depending on the PHU and surveillance period (Dussault et al. 2018).

### Mosquito Abundance Data and Preprocessing

The primary dependent variable in our forecasting models is Weekly Mosquito Abundance (WMA), defined as the mean catch per trap-night following reporting standards from Public Health Ontario (Public Health Ontario 2023). For each PHU and epidemiological week (Sunday to Saturday), WMA for *Cx. pipiens/restuans* was calculated as the total number of mosquitoes collected divided by the total number of active trap-nights in that PHU-week.

The analytical unit in the present study was the PHU-week. Differences in sampling effort were incorporated into the calculation of WMA by dividing the total number of *Cx*. *pipiens/restuans* collected by the corresponding total number of active trap-nights within each PHU-week. This standardization accounts for variation in the amount of trapping effort but does not eliminate heterogeneity associated with trap placement, local habitat, or other PHU-specific surveillance characteristics.

The target variable was defined as weekly WMA values aggregated at the PHU level from 2002 to 2023, encompassing both the model development (2002 to 2021) and independent testing (2022 to 2023) periods. Although surveillance data were available for each PHU over multiple years, the number of observations per PHU was relatively limited (typically 300 to 400 PHU-weeks). Because the initial feature space included numerous lagged abundance and meteorological predictors, and because model evaluation required repeated data splitting, fitting separate machine-learning models for each PHU would likely yield less stable and less comparable performance estimates. For this reason, observations were pooled across PHUs for model development and validation.

To increase sample size for model development and validation, we aggregated WMA data across all 34 PHUs, resulting in 9,936 PHU-week observations. We used data from 2002 to 2021 (9,093 observations) for model development and reserved 2022 to 2023 (843 observations) as an independent test set. This aggregation strategy provided a larger and more stable basis for comparing multiple machine-learning methods.

WMA was strongly right-skewed and contained a very small number of exceptionally high PHU-week values. To limit the disproportionate influence of this extreme upper tail during model fitting, WMA was winsorized at 50 mosquitoes per trap-night before transformation. Values above 50 were replaced by 50; no observations were removed. Winsorization is a robust preprocessing procedure used to retain the full sample while bounding the leverage of extreme observations in statistical and machine-learning models (Sharma and Chatterjee 2021, Cheng and Young 2023).

The threshold was selected from the empirical distribution of WMA and affected only 10 of 9,093 observations in the 2002 to 2021 development dataset, approximately 0.11%. It therefore preserved the observed variation across more than 99.8% of the development observations while preventing a few values from exerting disproportionate influence on models fitted to the complete provincial dataset. This common preprocessing step also provided a bounded response scale for comparison across the three model classes.

Following winsorization, WMA was transformed as ln(1+WMA) to reduce right skew and then rescaled to the interval [0,1]. The resulting response represented abundance over the range from 0 to 50 mosquitoes per trap-night. Consequently, the models were designed to predict variation within this range and not to distinguish the magnitude of observed values above the winsorization threshold.

### Weather Data and Preprocessing

Previous studies have identified meteorological conditions as important drivers of mosquito abundance, particularly temperature, precipitation, humidity, wind speed, and solar radiation (Barker 2019, De Freitas Costa et al. 2024, Lu et al. 2024). These variables were therefore considered as candidate predictors in this study. Daily meteorological data were obtained from the ISIMIP3a observational climate forcing dataset based on ERA5 (Muñoz-Sabater et al. 2021, Fatolahzadeh Gheysari et al. 2024) and 20CRv3 reanalysis products (hereafter referred to as ISIMIP 20CRv3-ERA5). The ISIMIP3a 20CRv3–ERA5 data were used as spatially and temporally consistent historical meteorological forcing for retrospective model development and evaluation. ISIMIP3a observational climate forcing was developed principally for historical impact-model evaluation and attribution rather than for real-time weather forecasting. This dataset provides climate variables at a horizontal resolution of 0.5° and daily temporal resolution and has been widely used in climate impact and environmental modeling studies (Frieler et al. 2024). The data were downloaded from the ISIMIP data repository, covering the period 2002 to 2023. We used reanalysis data rather than station observations because WMA is spatially averaged from multiple trapping locations within each PHU, yet meteorological stations are not co-located with traps, and many PHUs lack stations with continuous records over the full study period. Reanalysis products therefore provide complete, spatially continuous, and temporally consistent coverage across all PHUs from 2002 to 2023.

The ISIMIP 20CRv3-ERA5 dataset integrates reanalysis with bias-adjustment procedures to improve consistency across variables and regions, making it suitable for long-term environmental and epidemiological analyses. Reanalysis-based meteorological datasets have been widely applied in WMA modeling and vector-borne disease risk assessment, including studies of WNV and other mosquito-borne pathogens (Tsantalidou et al. 2021, Marini et al. 2022). In this study, daily meteorological variables were spatially interpolated and aggregated to the PHU level prior to weekly aggregation for model development.

Weather data are distributed on a grid with a resolution of 0.5° longitude by 0.5° latitude, while PHUs are represented as polygons. To assign climate data to each unit, we employ spatial interpolation. This method involves calculating weighted averages from the intersecting grid cells within each unit. The process is carried out through the following steps:

1. Generate a gridded shapefile that encompasses the entire province.
2. Execute a GIS operation to identify the grids that intersect each PHU.
3. Compute the intersected areas for each grid that intersects with a PHU.
4. Allocate weights to all intersected grids proportional to the intersected area, ensuring that the sum of the weights equals 1 for each PHU.
5. Extract the weather data corresponding to the grid cells.
6. Utilize the information derived from steps 2 to 5 to interpolate weather data (weighted average) across all PHUs.

All spatial operations were implemented in ArcGIS Pro (Education Version) (Esri 2023). By applying spatial interpolation, we generated PHU-specific daily time series for 2002 to 2023 for the following weather variables: average temperature (TAS: °C), minimum temperature (TASMIN: °C), maximum temperature (TASMAX: °C), near-surface wind speed (SFCWIND: m/s), relative humidity (HURS: %), near-surface shortwave radiation (RSDS: W/m^2^), and daily precipitation was converted to mm/day as needed prior to weekly aggregation. These daily series were then aggregated to weekly values for subsequent model development. Specifically, weekly maximum temperature was defined as the maximum of daily TASMAX, weekly minimum temperature was defined as the minimum of daily TASMIN, and weekly precipitation (mm/week) was calculated as the sum of daily PR values over the same week. All other weekly variables (TAS, SFCWIND, RSDS, and HURS) were computed as weekly means of the corresponding daily values. Weekly weather data were calculated using epidemiological weeks to ensure consistency with the WMA data.

All meteorological variables were rescaled to a [0, 1] range using a min-max normalization process. For each variable, the minimum and maximum values were calculated from the 2002 to 2021 development dataset and then held fixed. The same scaling bounds were subsequently applied to the independent 2022 to 2023 test dataset, ensuring that observations from the test period did not influence the normalization parameters.

## Materials and methods

The methodology consisted of three main components. We first constructed the candidate feature space from lagged mosquito abundance and meteorological variables and then applied feature-selection procedures to reduce redundancy and identify informative predictor subsets. Next, we trained three predictive model classes based on the selected features. Finally, model performance was assessed using complementary validation schemes designed to evaluate overall accuracy, temporal generalizability, and spatial transferability.

### Feature Construction and Feature Selection

The response variable was defined as target-week WMA, denoted $WMA_{0}$. For the exploratory associations shown in Fig. 1, WMA and meteorological variables were retained on their original scales. For feature selection and subsequent model fitting, WMA refers to the winsorized, log-transformed, and min-max rescaled abundance used for model fitting. Let lag j denote the value observed j epidemiological weeks prior to the prediction week, where j=0 corresponds to the prediction week itself. Accordingly, the candidate predictor set comprised lagged mosquito abundance and lagged meteorological variables. Specifically, abundance predictors were $WMA_{j}$ for j=1,…,4, and meteorological predictors were $TAS_{j}$, $\mathrm{TASMI}N_{j}$, $\mathrm{TASMA}X_{j}$, $PR_{j}$, $\mathrm{SFCWIN}D_{j}$, $\mathrm{HUR}S_{j}$, and $\mathrm{RSD}S_{j}$ for j=0,…,6. The initial candidate feature space therefore contained 53 predictors. The predictor set was designed to capture two key sources of information for week-ahead forecasting: recent mosquito abundance, reflecting short-term population persistence, and lagged meteorological conditions, reflecting delayed effects on mosquito development, survival, and habitat. Abundance predictors were restricted to lags 1 to 4, corresponding to the 2 to 4 week generation time of *Culex* mosquitoes under typical summer conditions (Lebl et al. 2013), so that prior weeks’ abundance provides a direct proxy for the parent population contributing to current abundance. Meteorological predictors were considered at lags 0 to 6 to capture both concurrent and antecedent effects on habitat formation, aquatic development, and adult emergence (Baril et al. 2023). Inclusion of lag-0 meteorological variables represents weather conditions during the target week. These variables were retained during the research-development stage to assess the potential contribution of target-week weather to week-ahead prediction. Because historical reanalysis data were used in the present retrospective analysis, operational implementation will require short-term weather forecasts available at the time of prediction. The contribution of lag-0 variables in an operational setting will therefore depend on the availability and accuracy of those forecasts.

**Fig. 1.**
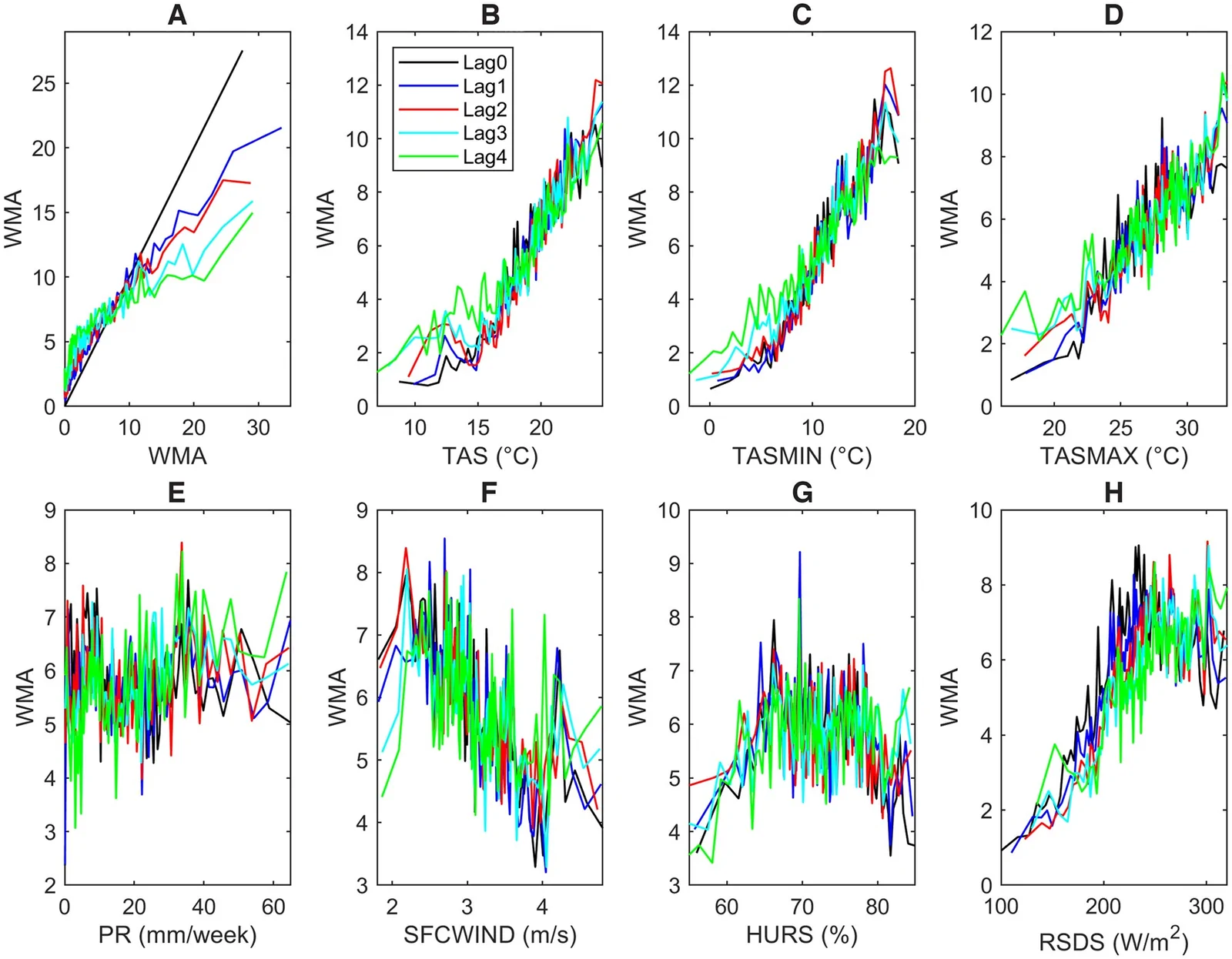
The relationship between WMA and other variables at different time lags (Lag0, Lag1, … Lag4) across Ontario’s PHUs. The variables include: (A) WMA; (B) TAS; (C) TASMIN; (D) TASMAX; (E) PR; (F) SFCWIND; (G) HURS; and (H) RSDS. For panel (A), Lag0 is shown as a reference only; abundance predictors used for forecasting were restricted to lags 1 to 4.

Meteorological records were complete across all specified lag windows. PHU-week observations were excluded whenever one or more of the four required lagged WMA values were unavailable. Most exclusions occurred during the first weeks of seasonal surveillance, when sufficient prior WMA history had not yet accumulated. The same complete-case rule was applied to mid-season observations when interruptions in surveillance produced an incomplete four-week WMA history. After filtering, 5,973 complete instances from 2002 to 2021 were retained for feature selection and model training, and 544 complete instances from 2022 to 2023 were retained for independent testing.

Before feature selection, exploratory associations between target-week WMA and the candidate predictors were visualized on their original scales to examine their general functional forms. Because raw predictor–response scatter plots showed substantial dispersion and overplotting, observations for each predictor–lag combination were ordered by predictor value and grouped into approximately 100 observations, with the final group retained only if it contained at least 50 observations. Mean predictor and WMA values were then calculated for each group and plotted.

This binned-mean procedure was used only for exploratory visualization and was not used in predictor selection, model fitting, or validation. Because binning smooths the data and may increase the apparent strength of associations, correlations calculated from the binned values were interpreted descriptively rather than inferentially.

Because candidate predictors were correlated across variables and temporal lags, feature selection was required to reduce redundancy and to identify a parsimonious subset of informative predictors. Numerous feature selection approaches have been proposed for predictive modeling, each emphasizing different criteria for assessing predictor relevance (Braun and Oswald 2011, Smith 2018, Theng and Bhoyar 2024). In this study, we applied three complementary feature selection approaches: stepwise regression (SR), the least absolute shrinkage and selection operator (LASSO), and random forest (RF), thereby reducing dependence on any single selection method.

SR is a wrapper-based method that iteratively adds and removes predictors to optimize a specified model selection criterion (Desboulets 2018). LASSO is a penalized regression approach that promotes sparse solutions by shrinking coefficients toward zero, thereby excluding weak predictors and reducing overfitting relative to stepwise procedures. RF provides a model-based measure of feature importance by quantifying the increase in out-of-bag (OOB) mean squared error resulting from permutation of each predictor, reflecting the loss of predictive information attributable to that feature (Iranzad and Liu 2025).

All feature-selection procedures were conducted using the 2002 to 2021 development dataset only and implemented in MATLAB R2024b. SR was performed using the *stepwiselm* function with the sum of squared errors (SSE) as the selection criterion, using bidirectional stepwise selection starting from a null (constant-only) model. LASSO regression was conducted using the *lasso* function, with the regularization parameter selected by minimizing 10-fold cross-validated mean squared error. RF models were implemented using MATLAB’s *TreeBagger* in regression mode with 50 trees, as preliminary analyses showed that OOB error decreased rapidly with increasing tree number and plateaued beyond approximately 50 trees. Permutation importance was quantified as the increase in OOB MSE after permuting each predictor. The predictor subsets identified by SR, LASSO, and RF were treated as alternative candidate feature sets and were subsequently evaluated across all three predictive model classes rather than being paired exclusively with the corresponding modeling approach.

### Model Selection and Training

We evaluated three regression-based modeling approaches to predict WMA: a stepwise multiple linear regression (SMLR) model which is a linear regression with interaction terms, a feedforward neural network (NN), and random forest regression (RFR). These models were selected to represent three commonly used and complementary predictive approaches in ecological forecasting: an interpretable linear model, a flexible nonlinear model, and an ensemble-based model. The aim was not to compare all possible machine-learning algorithms, but to assess a parsimonious set of representative methods for forecasting mosquito abundance within a common analytical framework. SMLR was included as an interpretable linear modeling approach for comparison with the nonlinear machine-learning models.

All models were implemented using the Statistics and Machine Learning Toolbox in MATLAB R2024b (Desboulets 2018). Unless otherwise specified, default parameter settings were retained to ensure a consistent comparison across model classes. Preliminary sensitivity checks indicated that predictive performance was relatively stable under moderate changes in key parameters, such as network size for NN and tree number for RFR. Accordingly, extensive hyperparameter tuning was not performed, and the comparison focused on differences in predictor information.

The SMLR model was implemented using MATLAB’s stepwiselm function. Candidate terms included main effects and two-way interactions among predictors. Stepwise selection was based on the SSE, and model parameters were estimated by ordinary least squares. Predictor entry and removal were determined by *P*-values associated with the corresponding F-statistic. This model provided an interpretable coefficient-based benchmark for assessing linear and interaction effects.

The NN model was implemented using MATLAB’s feedforwardnet function with two hidden layers containing 5 and 2 neurons, respectively. All remaining training settings were retained at default values unless otherwise specified. This configuration was used to provide sufficient flexibility to capture potentially nonlinear relationships between environmental drivers and mosquito abundance while avoiding unnecessary architectural complexity.

RFR was implemented using MATLAB’s TreeBagger function in regression mode, which constructs an ensemble of decision trees using bootstrap aggregation (Cutler et al. 2007). The ensemble included 50 regression trees, as preliminary analyses indicated that out-of-bag error decreased rapidly with increasing tree number and stabilized beyond approximately 50 trees. All remaining hyperparameters were retained at default values. This model was included because it can capture nonlinear responses and higher-order interactions while providing stable predictions.

### Model Evaluation

We evaluated predictive performance using three complementary validation schemes, each addressing a different aspect of model generalizability: (i) 10-fold cross-validation to estimate overall accuracy under random partitioning (Dutschmann et al. 2023, Le et al. 2024), (ii) leave-one-year-out validation to test temporal generalizability to unseen years (Sreedharan et al. 2023), and (iii) leave-one-PHU-out validation to assess spatial transferability to PHUs not used for training. In each leave-one-PHU-out iteration, all observations from the held-out PHU were excluded from model training. This grouped validation design evaluated transferability to an unseen PHU while preserving the PHU-level grouping of repeated observations (Roberts et al. 2017). This grouped validation design assesses spatial transferability but does not explicitly model within-PHU correlation or PHU-level random effects. A persistence model was included as the reference benchmark, with next-week abundance predicted from the immediately preceding week (${\mathrm{WMA}}_{0}={\mathrm{WMA}}_{1}$). This benchmark was used to assess whether the forecasting models provided predictive improvement beyond the most recently observed mosquito abundance.

For all validation strategies, predictive performance was quantified using the root mean square error (RMSE) and the coefficient of determination (*R²*), computed using only the held-out data. The RMSE is calculated using the formula:


(1) $$\mathrm{RMSE}=\sqrt{\frac{1}{N}\sum_{i=1}^{N}{\left(y_{i}-{\hat{y}}_{i}\right)}^{2}}.$$

And the *R²* value is calculated using the formula:


(2) $$R^{2}=1-\frac{\sum_{i=1}^{N}{\left(y_{i}-{\hat{y}}_{i}\right)}^{2}}{\sum_{i=1}^{N}{\left(y_{i}-\bar{y}\right)}^{2}}$$

where $y_{i}$ is the observed value, ${\hat{y}}_{i}$ is the predicted value, $\bar{y}$ is the mean of the observed values, and N is the number of observations. Because the response variable (WMA observations) was winsorized, log-transformed, and min-max normalized, model performance was assessed primarily on this transformed scale, which reduces the influence of right skew and extreme observations. For biological interpretation, RMSE is also reported on the original WMA scale (mosquitoes per trap-night), and back-transformed predictions compared with the original observed WMA values in Fig. 2. Predictions were converted back to the original WMA scale by reversing the min–max rescaling and log transformation, then


(3) $$\mathrm{WMA}=\exp(\ln\left(51\right)*{\hat{y}}_{i})-1.$$

**Fig. 2.**
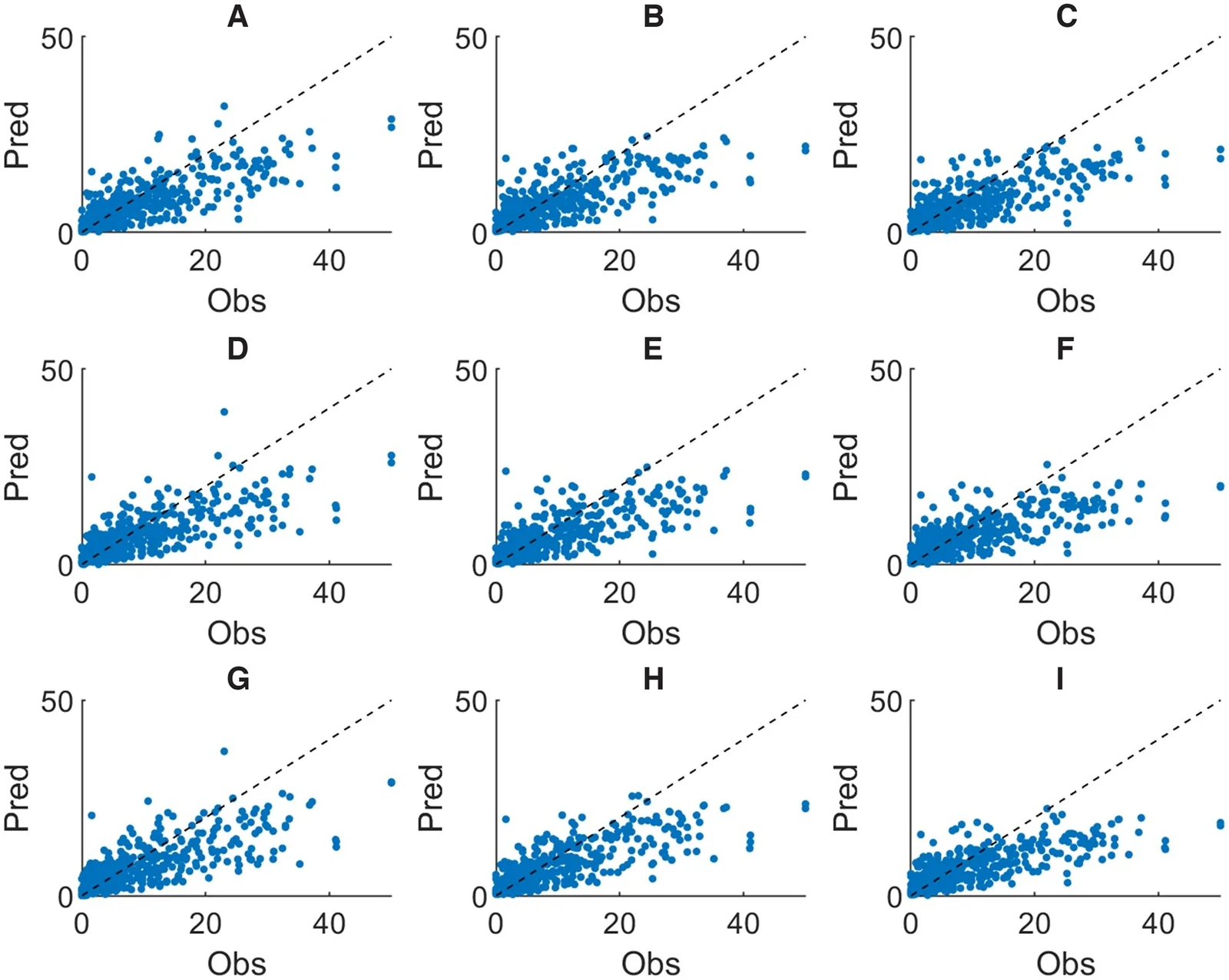
Back-transformed predicted versus original observed WMA on the independent 2022 to 2023 test set. Scatter plots compare predicted WMA (*y*-axis) with observed WMA (*x*-axis) for three predictor sets and three model types: type AC predictors with (A) SMLR, (B) NN, and (C) RFR; Type ABCD predictors with (D) SMLR, (E) NN, and (F) RFR; and Type E predictors with (G) SMLR, (H) NN, and (I) RFR. Each point represents a PHU-week observation. The dashed line indicates perfect agreement between predictions and observations (Predicted WMA = Observed WMA).

## Results

Previous studies have shown that WMA is associated with both recent WMA and meteorological conditions, including temperature, precipitation, humidity, and wind. Here, we provide an exploratory assessment of these associations to motivate the candidate predictor set and lag structure used in subsequent model development.

### WMA Statistics

Before developing predictive models, we first characterize the statistical properties of WMA. Specifically, we examine its distribution, seasonal patterns, temporal trends, and spatial variation across the province of Ontario.

Figure 3 presents the distribution of WMA based on 9,093 observations collected during 2002 to 2021. WMA ranged from 0.0 to 100.6 and was recorded to one decimal place. The distribution was highly right-skewed, with a mean of 5.81, and more than 2,000 observations fell within (0,1). Only 10 of the 9,093 development observations, approximately 0.11%, had WMA values ≥50. These observations were retained but winsorized at 50 before transformation and model fitting. The purpose of this preprocessing step was to limit the leverage of the very small extreme upper tail while preserving all PHU-week observations. Because values above 50 were assigned the threshold value during training, the fitted models should be interpreted as predicting WMA within the range of 0 to 50 rather than estimating the precise magnitude of exceptionally high observations.

**Fig. 3.**
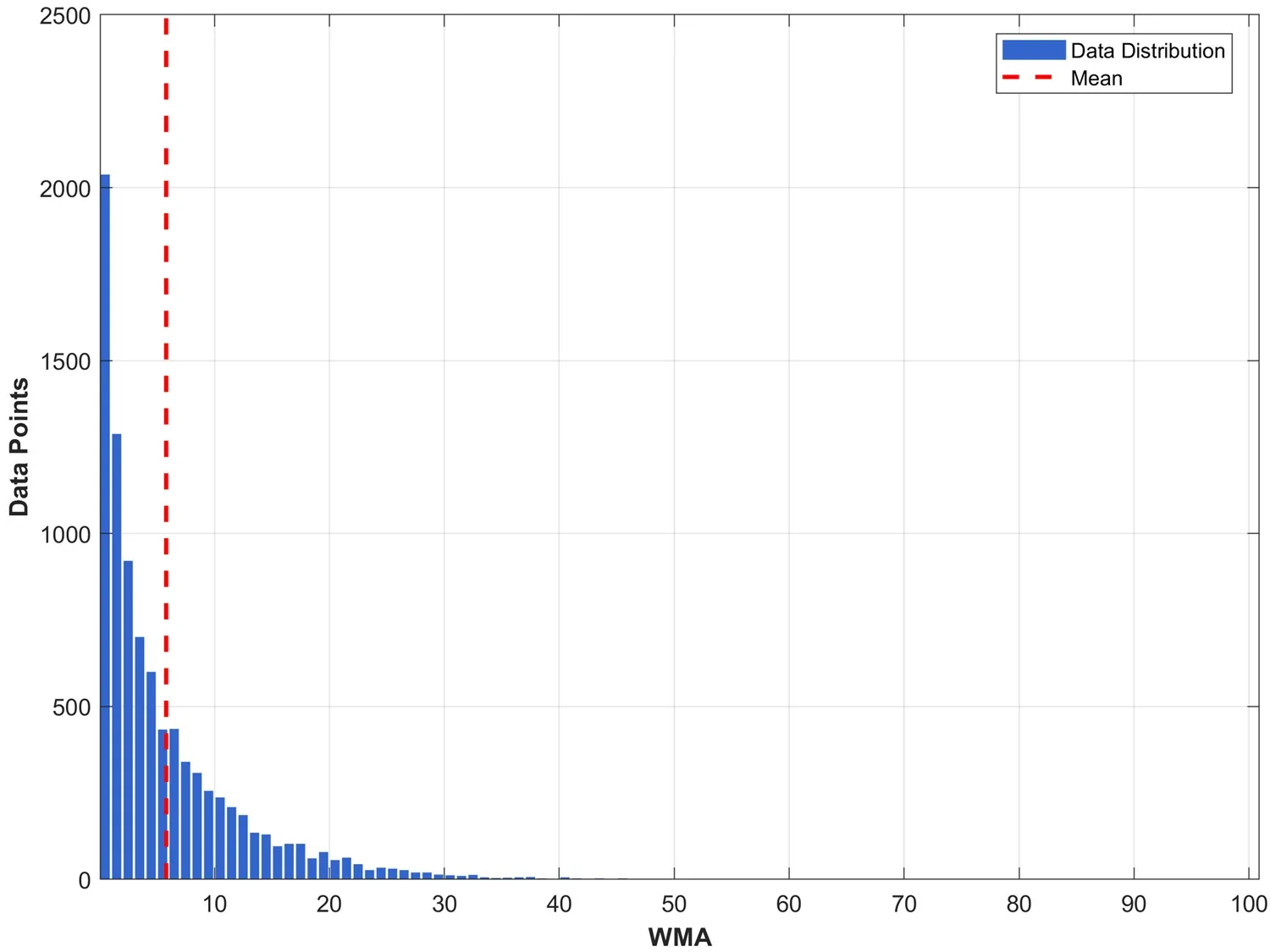
WMA in Ontario (2002 to 2021): Histogram based on observations from 34 Public Health Units across Ontario, Canada. The *x*-axis represents the intervals of WMA values, while the *y*-axis denotes the number of observations within each interval. The dashed vertical line indicates the mean value of all observations. The histogram bin width is 1.0.

To examine temporal variation in WMA, we summarized the data by epidemiological week and year. For the seasonal analysis, the median and maximum WMA were calculated for each epidemiological week across all PHUs and years. For the interannual analysis, the median and maximum WMA were calculated across all PHU-week observations within each year. These patterns are shown in Fig. 4. Figure 4A demonstrates a consistent seasonal peak in both median and maximum WMAs, typically occurring around the 30th to 33rd week of the year (late July to mid-August). This pattern indicates a strong seasonal trend, potentially driven by environmental factors such as temperature and rainfall, which favor mosquito breeding. Figure 4B presents the inter-annual variability in WMA over a 20-year period (2002 to 2021). The data does not reveal a significant trend in WMA over this period. However, some years, namely 2003, 2005, and 2013, displayed notably higher WMA, with 2013 recording particularly high median and maximum abundances. These fluctuations may be attributed to factors such as changes in public health practices.

**Fig. 4.**
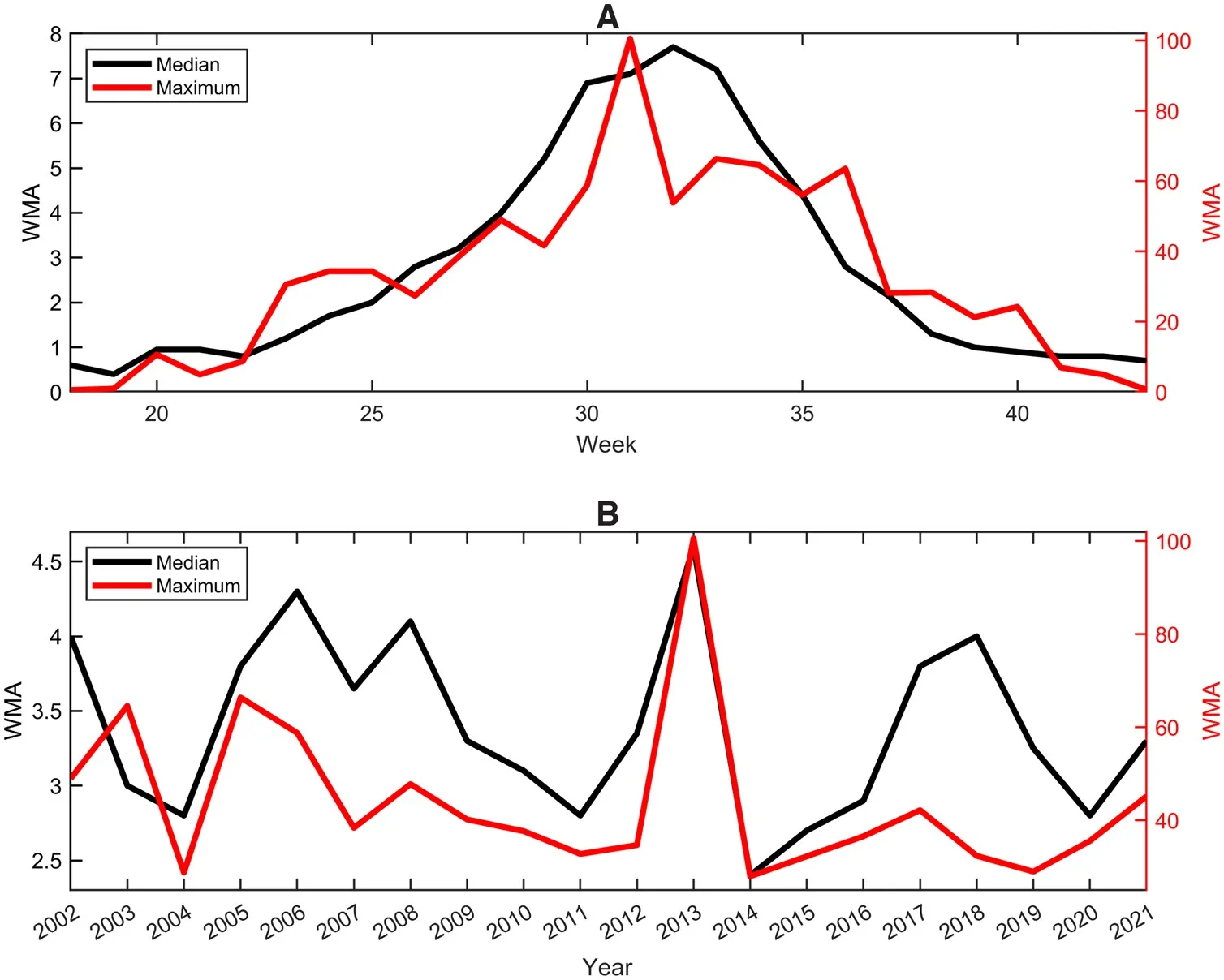
Temporal variation in WMA. (A) Seasonal fluctuation of WMA over a 26-week period within a year; (B) Annual variation in WMA from 2002 to 2021. In both sub-figures, the black line represents the median value, and the red line represents the maximum value. These values are derived from the WMA data collected from 34 Public Health Units across Ontario, Canada, during the period of 2002 to 2021, specifically from the 18th to the 43rd week of each year.

To discern the general spatial pattern of WMA in Ontario, we classified 20 years of WMA by PHU and computed both maximum and median values, as presented in Fig. 5 (Ontario Ministry of Natural Resources and Forestry 2023).

**Fig. 5.**
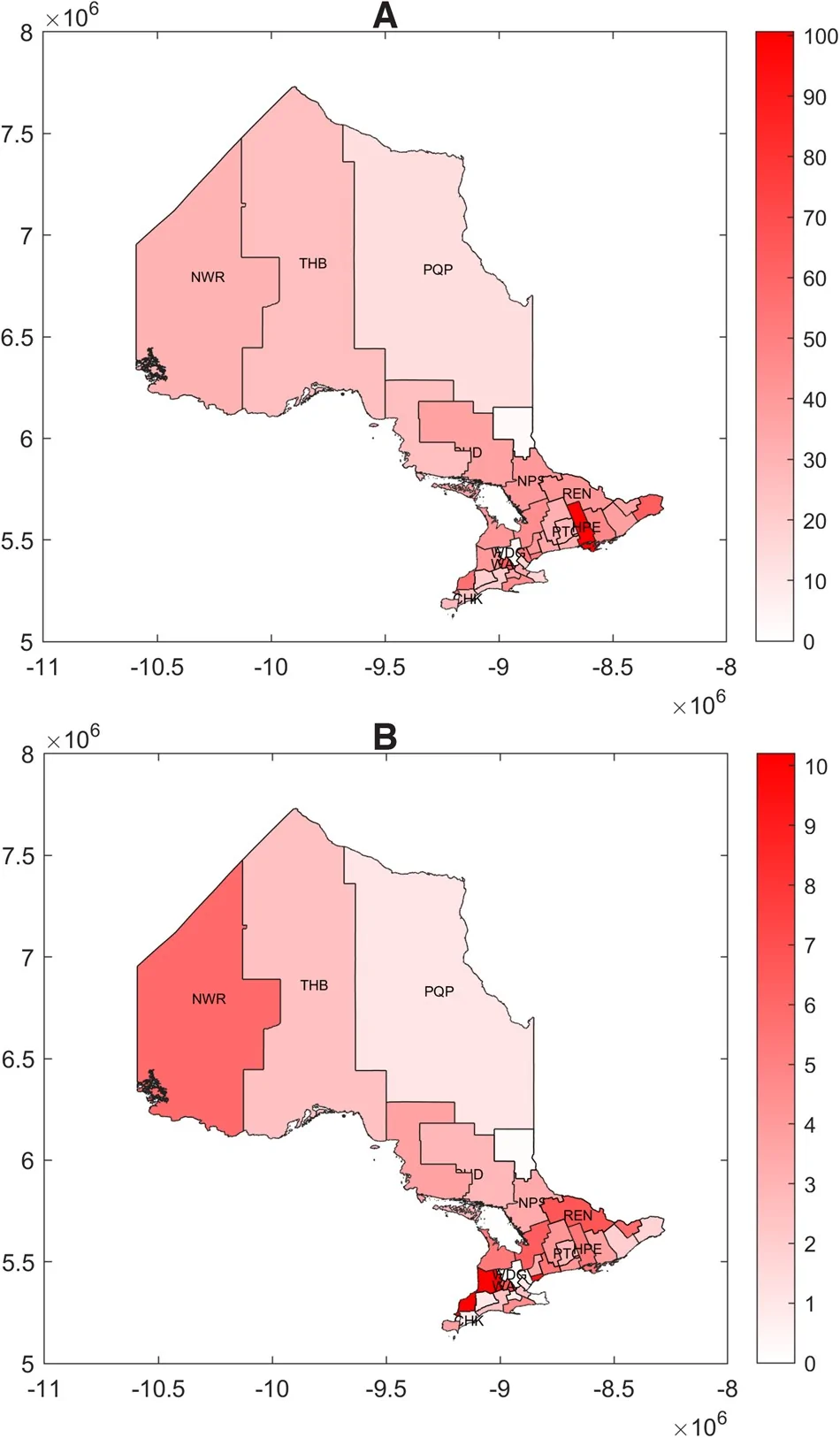
Spatial Distribution of WMA from 2002 to 2021. (A) Distribution of the maximum abundance values; (B) Distribution of the median abundance values. The units of *X* and *Y* coordinate are meters.

Figure 5 shows that the *Cx. pipiens/restuans* mosquito is distributed across the entire province. The southern regions of Ontario are characterized by higher maximum and median abundance values, whereas the northern regions display lower values. However, the spatial pattern does not exhibit a significant decrease with latitude or longitude. PHUs with high and low WMA values are distributed almost randomly. The maximum value was observed in the HPE unit in 2013 (Fig. 5A). An examination of spatial distribution maps (not shown) over the years reveals that both PQP and THB typically exhibit low abundance. Notably, in some years, the PQP unit shows an absence of mosquitoes. Environmental conditions, including temperature, humidity, and the availability of aquatic habitats, vary across the province and may be associated with the observed spatial variation in WMA.

### Exploratory Associations

We examined associations between the target-week mosquito abundance (${\mathrm{WMA}}_{0}$) and (i) lagged mosquito abundance ($WMA_{j}$, j=1,…,4) and (ii) weekly meteorological variables ($TAS_{j}$, $\mathrm{TASMI}N_{j}$, $\mathrm{TASMA}X_{j}$, $PR_{j}$, $\mathrm{SFCWIN}D_{j}$, $\mathrm{HUR}S_{j}$, and $\mathrm{RSD}S_{j}$, j=0,…,4); lags 0 to 4 are visualized in Fig. 1.

Overall, ${\mathrm{WMA}}_{0}$ showed strong positive association with recent mosquito abundance (${\mathrm{WMA}}_{1}-{\mathrm{WMA}}_{4}$), consistent with short-term population persistence (Fig. 1A). Temperature variables (TAS, TASMIN, TASMAX) exhibited positive associations with ${\mathrm{WMA}}_{0}$ at short lags (Fig. 1B-D), whereas precipitation showed relatively weak and variable associations across lags (Fig. 1E). Wind speed tended to be negatively associated with ${\mathrm{WMA}}_{0}$, consistent with reduced flight activity and trap catch under windy conditions (Fig. 1F). Humidity (Fig. 1G) and shortwave radiation (Fig. 1H) showed more nonlinear and context-dependent patterns. Across meteorological predictors, associations generally weakened with increasing lag. These exploratory patterns support the inclusion of recent WMA and short-lag weather variables as candidate predictors for the forecasting models developed below.

### Feature Engineering and Feature Selection

Based on the lag structure defined in Feature Construction and Feature Selection, the candidate feature space comprised 53 predictors drawn from recent mosquito abundance history and lagged meteorological covariates. To reduce redundancy among correlated variables and temporal lags, we applied three complementary feature-selection methods, namely SR, LASSO, and RF, and compared the resulting retained predictor subsets (Fig. 6).

**Fig. 6.**
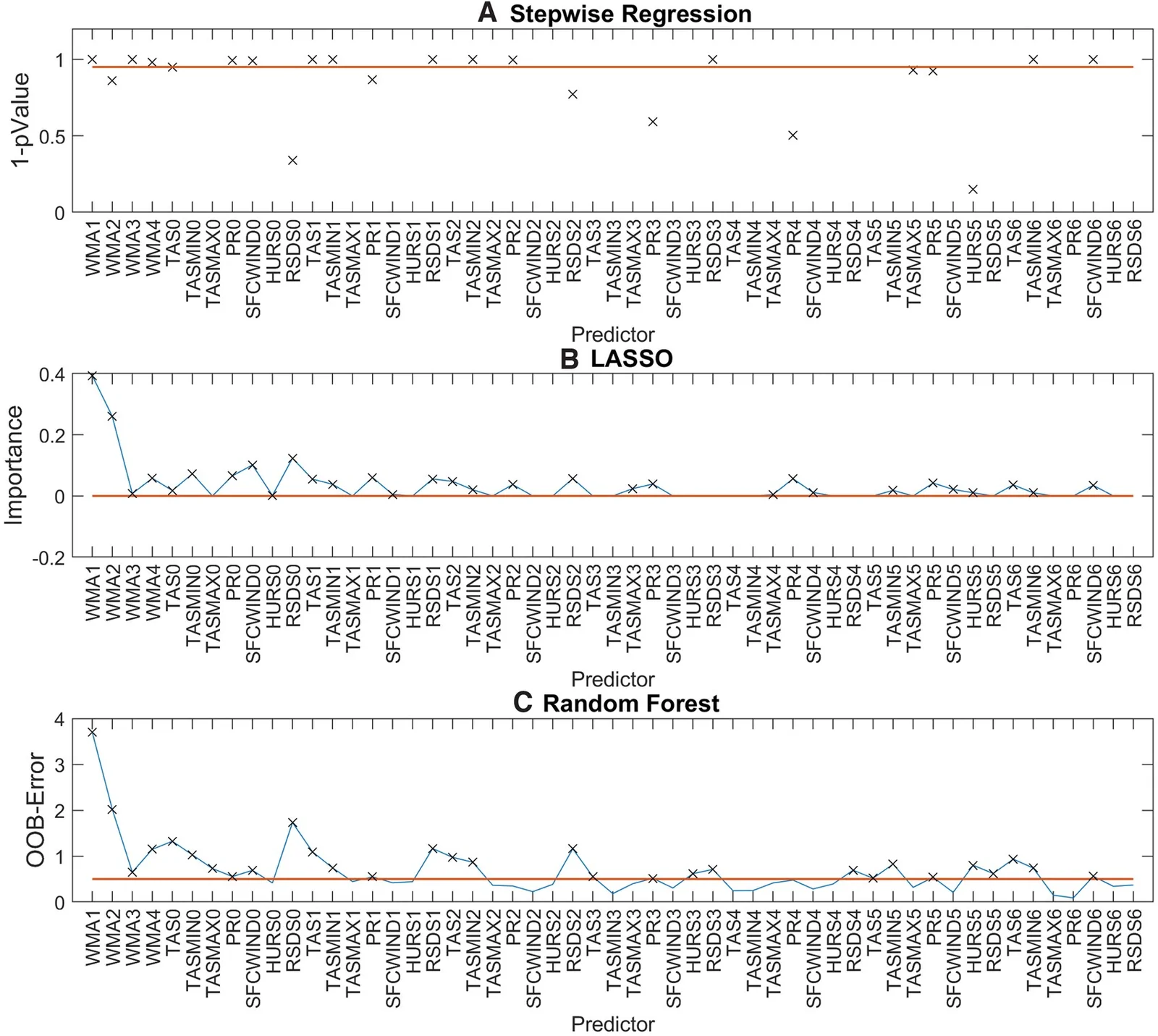
Variable importance under three feature selection methods: (A) SR (SSE-selected model); points show 1 − *P* values for retained linear terms as descriptive statistics, with the horizontal line at 1−*P* = 0.95 (*P* = 0.05) shown for reference only; (B) LASSO (coefficient magnitude); and (C) RF importance based on permutation-induced increases in OOB error. Red lines in panels (B) and (C) indicate the thresholds used to define retained predictors. The “×” symbol marks each predictor’s score.

Across the three feature-selection methods, a stable core subset of predictors was identified. Seventeen predictors were selected by all three methods, indicating substantial agreement on the main predictive signals. In total, 27 predictors were selected by at least two methods, 15 were selected by only one method, and 11 were not selected by any method. In Fig. 6A, some predictors appear retained despite weak marginal linear effects because associated interaction terms improved overall predictive performance under the SSE-based stepwise selection criterion; for clarity, only linear terms are shown in the figure.

Recent mosquito abundance was the most consistently retained predictor group, indicating that short-term population persistence provides the dominant signal for week-ahead forecasting. Among meteorological variables, predictors at lags 0 to 2 were retained most frequently, suggesting that immediate and recent weather conditions contribute additional short-term information beyond abundance history alone. Temperature-related variables and precipitation were among the most consistently retained weather predictors, which is ecologically plausible because mosquito development, habitat availability, and adult emergence respond rapidly to ambient conditions. Wind-related predictors were also retained in some models, consistent with effects on adult activity and trap catch.

In contrast, intermediate lags of 3 to 5 weeks were selected infrequently, whereas only a small number of longer-lag predictors were retained consistently. These longer-lag features may reflect delayed influences on population buildup or broader seasonal structure, but because weather variables are correlated across both type and lag, they should be interpreted cautiously. Overall, the feature-selection results indicate that predictive performance is driven primarily by a compact set of recent abundance and short-lag weather predictors, while additional lagged variables contribute comparatively little.

Finally, feature selection in this study was prediction-oriented rather than mechanistic. Predictors were retained on the basis of their contribution to predictive performance, and correlated predictors may therefore substitute for one another across methods. Accordingly, the next section evaluates multiple engineered predictor sets, including both the common core predictors and method-specific selections, to determine how these alternative feature sets affect forecasting accuracy.

### Model Performance and Validation

We evaluated 14 predictor sets: the base subsets A to D, their combinations (AB, AC, BC, CD, ABC, ACD, BCD, and ABCD) from the 17 common predictors, and the three method-specific sets (E to G). We grouped the predictors into the following types:

- Type A: WMA at different lags (${\mathrm{WMA}}_{1}-{\mathrm{WMA}}_{4}$);
- Type B: concurrent weather predictors ($TAS_{0}$, $PR_{0}$, $\mathrm{SFCWIN}D_{0}$, and $\mathrm{RSD}S_{0}$);
- Type C: near-term antecedent weather predictors at lags 1 to 2 ($TAS_{1}$, $\mathrm{TASMI}N_{1}$, $PR_{1}$, $\mathrm{RSD}S_{1}$, $\mathrm{TASMI}N_{2}$, and $\mathrm{RSD}S_{2}$);
- Type D: longer-lag weather predictors at lags 4 to 6 ($PR_{4}$, $\mathrm{TASMI}N_{6}$, and $\mathrm{SFCWIN}D_{6}$);
- Type E: all 23 predictors selected by the SR method;
- Type F: all 31 predictors selected by the LASSO method; and
- Type G: all 32 predictors selected by the RF method.

These ranged from autoregressive terms only (Type A: WMA lags 1 to 4) to comprehensive sets selected by SR (Type E), LASSO (Type F), and RF (Type G), all of which were evaluated across the three model architectures (SMLR, NN, and RFR). We assessed predictive power using three cross-validation (CV) strategies: 10-fold CV for overall accuracy, leave-one-year-out CV for temporal robustness, and leave-one-PHU-out CV for spatial generalizability.

Table 1 summarizes 10-fold cross-validation performance (RMSE and *R²*) for three model architectures (SMLR, NN, and RFR) across multiple predictor sets. Overall, predictive performance is dominated by recent WMA: autoregressive terms alone (Type A) explain approximately 60% of the variance in WMA. In contrast, models using only weather predictors (combinations of Types B-D) explain substantially less variance (*R*²  ∼ 0.30 to 0.49). Notably, antecedent weather (Type C) was consistently more informative than concurrent weather (Type B), indicating that delayed meteorological effects were more relevant to WMA prediction than same-week conditions.

When autoregressive terms are combined with weather variables, model performance improves markedly. Predictor sets that include WMA lags together with concurrent and near-term weather (eg AB, AC, ABC, ACD, and ABCD) consistently achieve the highest *R*² (∼ 0.69) and lowest RMSE (∼ 0.13). Importantly, performance differences among these top-performing predictor sets are small (typically ≤0.003 for RMSE and ≤0.01 for *R*²), indicating that model skill is driven by a stable core signal—recent WMA plus short-lag weather—while additional predictors yield diminishing returns.

The consensus predictor set (Type ABCD; 17 predictors shared across all three feature-selection methods) performs essentially as well as the larger method-specific sets (Types E–G), for which improvements are negligible (<0.3% in explained variance). This pattern is consistent with limited overfitting and suggests that the consensus set captures the key predictive information while reducing model complexity. From an operational standpoint, Type ABCD is therefore preferred because it provides near-maximal accuracy with fewer predictors and a simpler, more robust data pipeline.

Across modeling approaches, RFR model generally provides the best or near-best performance across predictor sets, whereas SMLR and NN perform similarly but slightly worse in many configurations. However, the differences among model architectures are minimal for the best-performing predictor sets, further supporting the robustness of the forecasting framework.

To assess operational reliability over time, we evaluated model performance using leave-one-year-out validation. Results were broadly consistent across predictor sets ABCD and E–G, with only minor differences in performance. As an illustrative example, Fig. 7 presents results for the Type ABCD predictor set.

**Fig. 7.**
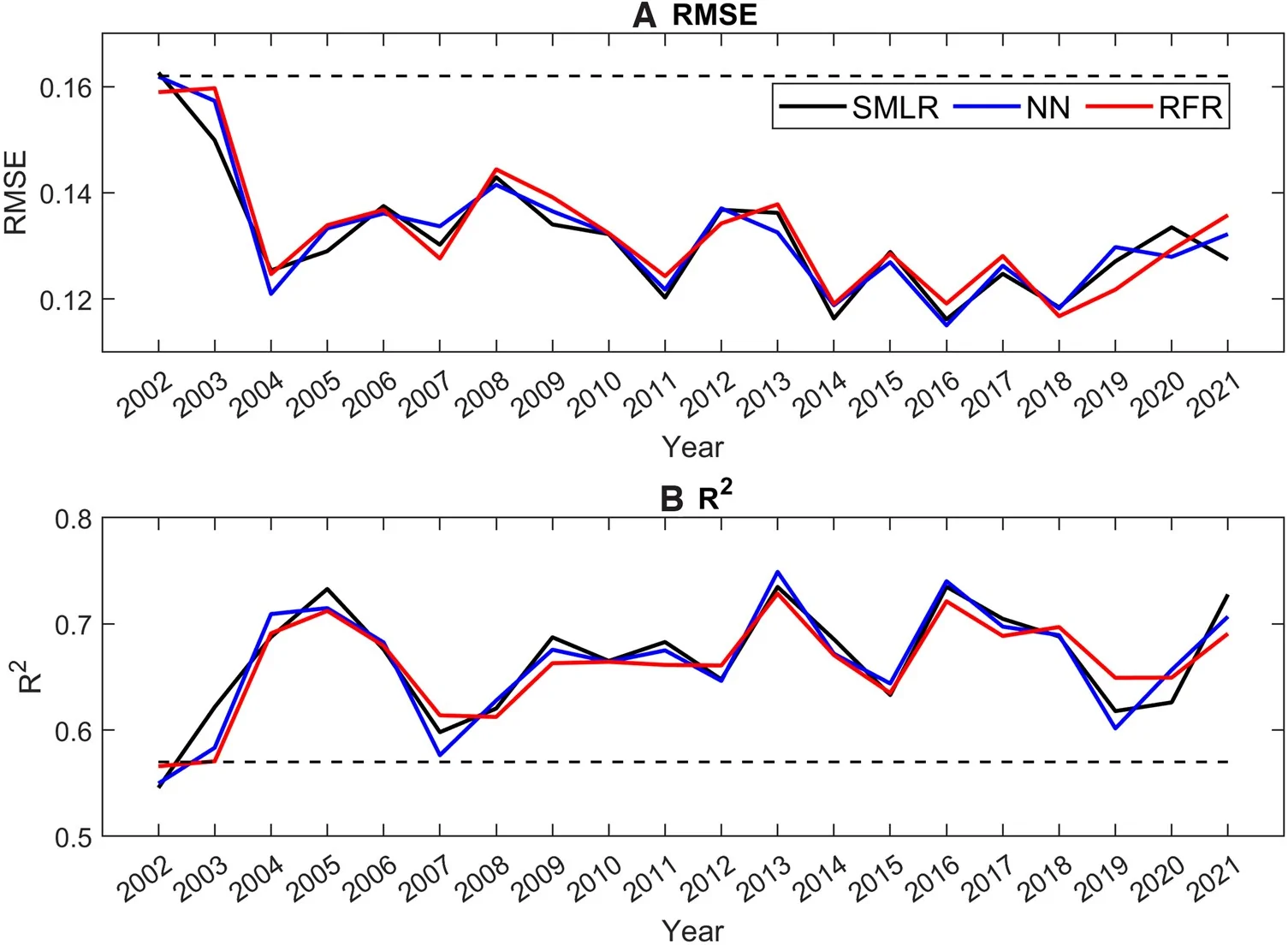
Leave-one-year-out cross-validation performance for models trained using the Type ABCD predictor set on the training data set. (A) RMSE and (B) *R²* computed on the held out (test) year. In both panels, the horizontal dashed line indicates the persistence baseline, defined as $WMA_{0}$ = $WMA_{1}$, i.e. next week’s mosquito abundance is predicted by the current week’s value. This baseline yielded an RMSE of 0.16 and an $R^{2}$ of 0.57.

Overall, model performance was temporally stable after 2003, with *R²* values typically ranging between 0.60 and 0.70, indicating consistent predictive skill across most years. Performance was particularly strong during years with high WMA (e.g. 2013 and 2018), as reflected by relatively low RMSE and high *R²* values, suggesting that the models capture conditions associated with elevated population levels effectively. By contrast, predictive accuracy was lower during the early years of the dataset (2002 to 2003), likely reflecting the initial phase of the surveillance program, when data coverage was more limited and variability was higher.

To evaluate spatial generalizability, we assessed model performance using leave-one-PHU-out validation. As an illustrative example, Fig. 8B presents results for the Type ABCD predictor set.

**Fig. 8.**
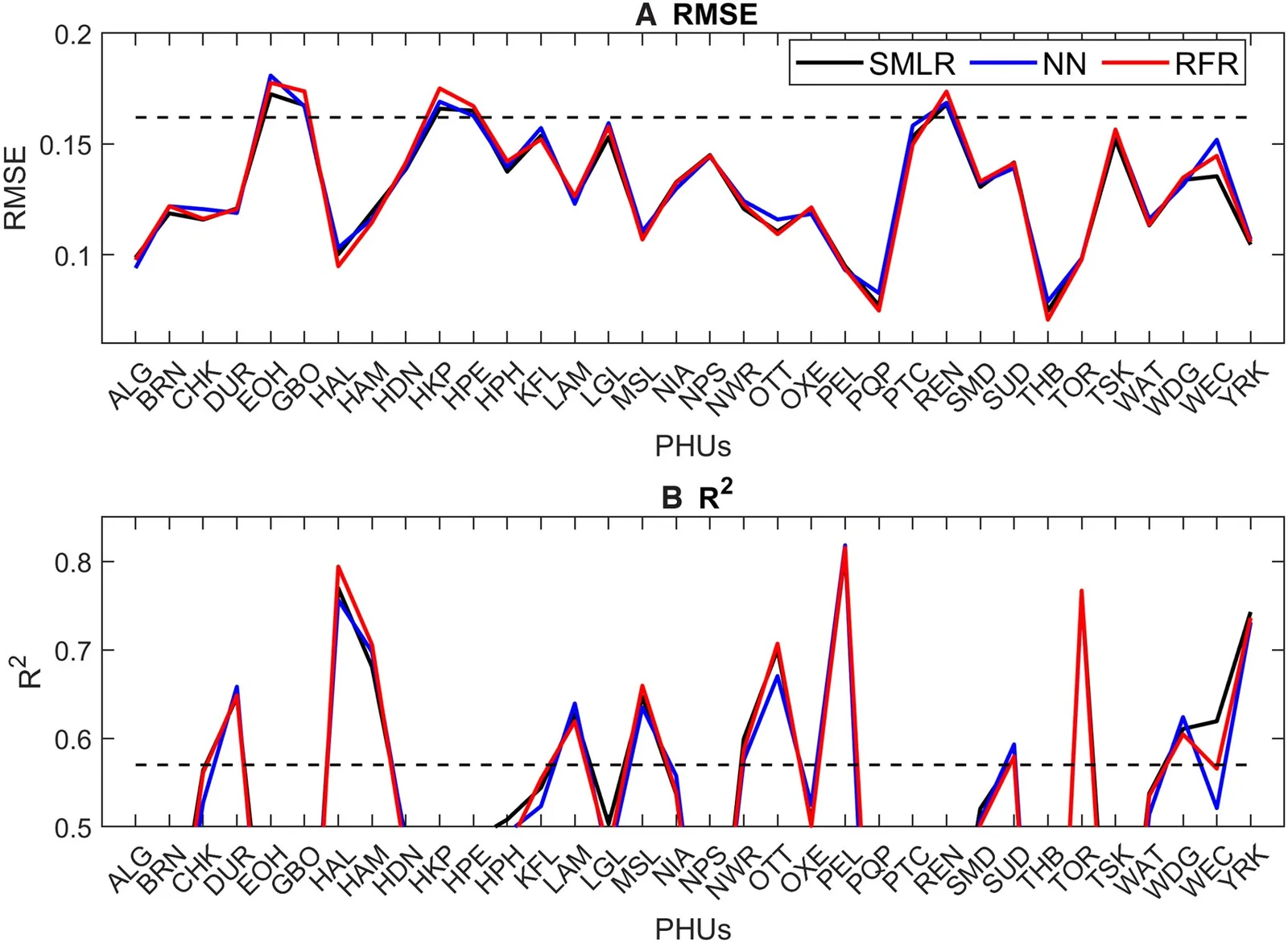
Leave-one-PHU-out validation performance on the development dataset. Panel (A) depicts the RMSE, and panel (B) illustrates the *R* ², both based on the data from the testing PHU. The horizontal dashed lines in the Fig. 7A, B represent the persistence baseline values of RMSE = 0.16 and *R* ² = 0.57, respectively.

Overall, models demonstrated strong performance in southern and central Ontario PHUs characterized by moderate to high WMA, including Peel (PEL), Toronto (TOR), and York (YRK), where more than 50% of the variance was typically explained. By contrast, predictive performance was lower in northern PHUs such as Porcupine (PQP), Thunder Bay (THB), and Timiskaming (TSK). This reduction in accuracy likely reflects the predominance of near-zero mosquito counts and limited abundance variability in these PHUs. Because these regions generally exhibited low *Cx. pipiens/restuans* abundance in the surveillance data, the practical consequences of reduced quantitative accuracy may be smaller than in higher-abundance PHUs. Nevertheless, predictions for these low-abundance regions should be interpreted cautiously.

### Independent Test Set Evaluation (2022 to 2023)

We used the 544 PHU-week observations from 2022 to 2023 as an independent test set to evaluate models trained on 2002 to 2021 using 7 predictor sets. To ensure comparability with the training period and to avoid information leakage, predictors in the 2022 to 2023 test set were rescaled using the minimum and maximum values computed from the 2002 to 2021 training data only. Overall, predictive performance was stable across predictor sets, with test-set $R^{2}\in [0.66,\;0.69]$ and RMSE≈0.14 on the rescaled log-transformed response scale. Because model fitting and primary evaluation were conducted on this transformed scale, Table 2 additionally reports RMSE on the original WMA scale (mosquitoes per trap-night) to quantify prediction error in the original biological units; on that scale, RMSE∈[5.8, 6.5]. Relative to the persistence baseline (RMSE = 0.16, $R^{2}=$0.57), the best-performing model reduced RMSE by 14.4% and increased by 0.124 on the independent 2022 to 2023 test set. These gains indicate additional predictive information beyond recent WMA alone, although the magnitude of improvement was moderate. The small differences among these predictor sets suggest that model skill is largely driven by a stable core signal—recent WMA and short-lag weather conditions—while additional predictors provide only marginal gains. From an operational perspective, the parsimonious 17-predictor set (Type ABCD) is therefore preferred because it achieves near-maximal accuracy while reducing model complexity and data requirements for routine forecasting. When complete weather data are not available, simplified predictor sets (AB, AC, and ABC) remain effective and offer practical alternatives for operational forecasting.

RMSE and *R*² values in Tables 1 and 2 are highly similar for corresponding predictor sets and models, indicating robust generalization and limited sensitivity to predictor or model choice. With adequate data, SMLR, NN, and RFR yield comparable predictive accuracy for WMA.

When predictions were transformed back to the original scale, agreement was greatest for the low-to-moderate WMA values that comprised most observations in the independent test set. Predictions were lower than several of the highest original WMA observations. This pattern should be interpreted in light of the preprocessing procedure: because training responses above 50 were winsorized at 50, the models were not designed to reproduce differences in magnitude among observations above that threshold. Figure 2 therefore evaluates general agreement across the observed abundance distribution but does not demonstrate accurate prediction of the most extreme abundance values.

Collectively, the feature-selection and validation results indicate limited sensitivity of forecasting performance to the exact composition of the predictor set, provided that recent WMA and short-lag meteorological variables are retained. Recent abundance history consistently provided the dominant predictive signal, whereas weather variables contributed smaller additional gains, mainly at lags 0 to 2. Intermediate lags were rarely selected, and larger feature sets improved performance only marginally relative to the parsimonious consensus set. These findings suggest that the week-ahead forecasting framework is robust to moderate variation in input specification.

## Discussion

We developed and evaluated week-ahead forecasting models for *Cx. pipiens/restuans* abundance across 34 Ontario PHUs using 22 years of surveillance data (2002 to 2023). Across all validation schemes, recent WMA at lags 1 to 4 consistently emerged as the dominant predictor of next-week abundance, whereas meteorological variables provided additional but secondary gains in predictive skill. Performance was similar across SMLR, NN, and RFR models, and the parsimonious 17-predictor consensus set performed comparably to larger predictor sets. This finding supports the operational value of relatively simple and interpretable models for short-term mosquito forecasting.

The importance of recent mosquito abundance is consistent with short-term population persistence, while the additional contribution of meteorological predictors agrees with previous *Culex* studies showing associations with antecedent temperature, precipitation, humidity, and wind conditions (Wang et al. 2011, Lebl et al. 2013, Simonetto et al. 2026). Lagged environmental conditions have also been shown to support short-term prediction of *Cx. pipiens/restuans* population dynamics (Ripoche et al. 2019). Our finding that recent and short-lag predictors contributed most strongly is therefore consistent with previous evidence that mosquito abundance reflects both recent population levels and antecedent environmental conditions.

The limited gains from increasing model complexity and predictor-set size suggest that a relatively parsimonious forecasting framework can balance predictive accuracy with implementation requirements (Johnson-Bice et al. 2021, Scavia et al. 2021). A recent mosquito-forecasting study similarly found that a simpler generalized linear model outperformed RF on held-out test data despite the superior fit of RF to the training data, indicating that greater model complexity does not necessarily improve out-of-sample prediction (Steindorf et al. 2025). These findings support the operational value of a relatively parsimonious forecasting framework.

Validation results demonstrated robust generalization across both temporal and spatial holdouts, and independent testing in 2022 to 2023 data confirmed reliable out-of-sample performance. Model performance was interpreted not only using *R*² and RMSE but also relative to the persistence benchmark. Although the forecasting models improved upon persistence, the incremental gain was moderate, and performance remained lower for unusually high abundance and some low-abundance PHUs. In several northern PHUs, lower predictive accuracy coincided with relatively low WMA and limited abundance variability. Previous research in eastern Ontario has also documented substantial spatial variation in *Cx. pipiens/restuans* habitat suitability and abundance patterns (Talbot et al. 2023). These findings suggest that local ecological conditions and surveillance characteristics should be considered when interpreting the spatial applicability of the forecasting framework.

Several limitations should be noted. Although feature selection and multiple validation schemes were used to reduce overfitting and assess generalizability, prediction uncertainty remains. The framework does not explicitly account for PHU-level effects or trap-level characteristics, which may contribute to residual spatial heterogeneity. In addition, because the models were developed using Ontario surveillance data, their transferability to other regions and surveillance systems requires further evaluation. Meteorological inputs were derived from reanalysis data rather than operational forecast products, and the present study represents the methodological development stage of an operational forecasting tool rather than a fully implemented real-time system. Because lag-0 meteorological predictors represent target-week conditions, their operational utility will also depend on the availability and accuracy of short-term weather forecasts at the time predictions are issued. Translating predictive mosquito models into routine public-health decision support remains an important challenge (Barker 2019), and implementation of the present framework will require further collaboration with public-health partners to establish forecast timing and integrate routinely available surveillance and meteorological inputs. The models also tended to underpredict unusually high abundance values. In operational use, such underprediction could delay recognition of elevated mosquito activity. Peak abundance has been an important challenge in previous *Cx. pipiens/restuans* modeling; Shutt et al. (2022) found that a process-based model incorporating water-level data captured year-to-year variation in peak abundance better than statistical models. In the present study, winsorization reduced the influence of a very small number of exceptionally high observations but limited differentiation among values above 50 mosquitoes per trap-night. Prediction of rare extreme abundance therefore remains an important direction for future work.

## Conclusion

The public-health significance of this work lies in its ability to provide reliable short-term forecasts of vector abundance using routinely collected surveillance and weather data. Such forecasts can improve situational awareness, support earlier identification of periods of elevated transmission risk, and help guide targeted mosquito control and surveillance efforts. By informing more efficient allocation of public-health resources, this framework strengthens operational preparedness and response capacity for WNV risk management in Ontario and potentially in other temperate regions with comparable surveillance systems.

## Supplementary Material

tjag159_Supplementary_Data

## Data Availability

Mosquito surveillance data used in this study are available from Public Health Ontario upon reasonable request. Meteorological data are publicly available and can be downloaded from the data source referenced in the manuscript.

## Code Availability

The code used for data processing, model development, and analysis is available from the authors upon reasonable request.
