# Supplementary material for: Forecasting Culex pipiens/restuans abundance in Ontario, Canada using surveillance and weather data
Source: J Med Entomol. 2026 Nov 5;63(6):tjag159. doi: 10.1093/jme/tjag159 (PMC13743428; doi:10.1093/jme/tjag159)
Supplement: Supplementary file 1 [file tjag159_supplementary_data.docx]

**Forecasting *Culex pipiens/restuans* Abundance in Ontario, Canada Using Surveillance and Weather Data**

Zhaowan Li, Ziwang Deng, Huaiping Zhu*

**Appendix**

**Table A1**. Summary of abbreviations.

| **Abbreviation** | **Full term** | **Source** |
| --- | --- | --- |
| WNV | West Nile virus | (Talbot et al. 2023) |
| PHU | Public Health Unit | (Talbot et al. 2023) |
| WMA | Weekly Mosquito Abundance | (This study) |
| ISIMIP | Inter-Sectoral Impact Model Intercomparison Project | (ISIMIP 2023) |
| SMLR | Stepwise Multiple Linear Regression | (Desboulets 2018) |
| NN | Neural Network | (Hoyos et al. 2021) |
| RFR | Random Forest Regression | (Desboulets 2018) |
| TAS | Near-Surface Air temperature | (ISIMIP 2023) |
| TASMIN | Daily Minimum Near-Surface Air temperature | (ISIMIP 2023) |
| TASMAX | Daily Maximum Near-Surface Air temperature | (ISIMIP 2023) |
| SFCWIND | Near-Surface Wind Speed | (ISIMIP 2023) |
| HURS | Near-Surface Relative Humidity | (ISIMIP 2023) |
| RSDS | Surface Downwelling Shortwave Radiation | (ISIMIP 2023) |
| PR | Precipitation | (ISIMIP 2023) |
| ERA5 | ECMWF Reanalysis v5 | (ISIMIP 2023) |
| 20CRv3 | Twentieth Century Reanalysis version 3 | (ISIMIP 2023) |
| SR | Stepwise Regression | (Desboulets 2018) |
| LASSO | Least Absolute Shrinkage and Selection Operator | (Hoyos et al. 2021) |
| RF | Random Forest | (Hoyos et al. 2021) |
| RMSE | Root Mean Squared Error | (Chen et al. 2019) |
| *R²* | Coefficient of Determination | (Dutschmann et al. 2023) |
| SSE | Sum of Squared Errors | (Braun and Oswald 2011) |
| OOB | Out-Of-Bag | (Iranzad and Liu 2025) |
| GIS | Geographic Information System | (Muñoz-Sabater et al. 2021) |

**Table A2**. PHUs in Ontario grouped by geographical region.

| **Geographical Region** | **Abbreviation** | **Official Public Health Unit Name** |
| --- | --- | --- |
| Northern | ALG | Algoma District |
|  | NPS | North Bay Parry Sound District |
|  | PQP | Porcupine |
|  | SUD | Sudbury and District |
|  | TSK | Timiskaming |
|  | NWR | Northwestern |
|  | THB | Thunder Bay District |
| Western | BRN | Brant County |
|  | HAL | Halton Regional |
|  | HAM | City of Hamilton |
|  | HDN | Haldimand-Norfolk |
|  | NIA | Niagara Regional Area |
|  | WAT | Waterloo |
|  | WDG | Wellington-Dufferin-Guelph |
|  | CHK | Chatham-Kent |
|  | GBO | Grey Bruce |
|  | LAM | Lambton |
|  | MSL | Middlesex-London |
|  | OXE | Oxford Elgin St. Thomas |
|  | HPH | Huron Perth |
|  | WEC | Windsor-Essex County |
| Central | DUR | Durham Regional |
|  | HKP | Haliburton-Kawartha-Pine Ridge District |
|  | PEL | Peel Regional |
|  | PTC | Peterborough County-City |
|  | SMD | Simcoe Muskoka District |
|  | TOR | City of Toronto |
|  | YRK | York Regional |
| Eastern | EOH | Eastern Ontario |
|  | HPE | Hastings and Prince Edward Counties |
|  | KFL | Kingston-Frontenac and Lennox and Addington |
|  | LGL | Leeds-Grenville and Lanark District |
|  | OTT | City of Ottawa |
|  | REN | Renfrew County and District |

**References**

Braun MT, Oswald FL. 2011. Exploratory regression analysis: A tool for selecting models and determining predictor importance. Behav Res. 43(2):331–339. https://doi.org/10.3758/s13428-010-0046-8

Chen S, Whiteman A, Li A, et al. 2019. An operational machine learning approach to predict mosquito abundance based on socioeconomic and landscape patterns. Landscape Ecol. 34(6):1295–1311. https://doi.org/10.1007/s10980-019-00839-2

Desboulets LDD. 2018. A Review on Variable Selection in Regression Analysis. Econometrics. 6(4):45. https://doi.org/10.3390/econometrics6040045

Dutschmann T-M, Kinzel L, Ter Laak A, et al. 2023. Large-scale evaluation of k-fold cross-validation ensembles for uncertainty estimation. J Cheminform. 15(1):49. https://doi.org/10.1186/s13321-023-00709-9

Hoyos W, Aguilar J, Toro M. 2021. Dengue models based on machine learning techniques: A systematic literature review. Artificial Intelligence in Medicine. 119:102157. https://doi.org/10.1016/j.artmed.2021.102157

Iranzad R, Liu X. 2025. A review of random forest-based feature selection methods for data science education and applications. Int J Data Sci Anal. 20(2):197–211. https://doi.org/10.1007/s41060-024-00509-w

ISIMIP. 2023. Search the ISIMIP Repository. ISIMIP Data Portal. https://data.isimip.org/search/tree/ISIMIP3a/InputData/climate/atmosphere/20crv3/obsclim/tas/.

Muñoz-Sabater J, Dutra E, Agustí-Panareda A, et al. 2021. ERA5-Land: a state-of-the-art global reanalysis dataset for land applications. Earth Syst. Sci. Data. 13(9):4349–4383. https://doi.org/10.5194/essd-13-4349-2021

Talbot B, Kulkarni MA, Rioux-Rousseau M, et al. 2023. Ecological Niche and Positive Clusters of Two West Nile Virus Vectors in Ontario, Canada. EcoHealth. 20(3):249–262. https://doi.org/10.1007/s10393-023-01653-8
